# Artificial intelligence for disease X: Progress and challenges

**DOI:** 10.1515/jtim-2024-0035

**Published:** 2025-01-10

**Authors:** Keda Chen, Jiaxuan Li, Lanjuan Li

**Affiliations:** Key Laboratory of Artificial Organs and Computational Medicine in Zhejiang Province, Shulan International Medical College, Zhejiang Shuren University, Hangzhou, Zhejiang Province, China; State Key Laboratory for Diagnosis and Treatment of Infectious Diseases, National Clinical Research Center for Infectious Diseases, National Medical Center for Infectious Diseases, Collaborative Innovation Center for Diagnosis and Treatment of Infectious Diseases, The First Affiliated Hospital, Zhejiang University School of Medicine, Hangzhou, Zhejiang Province, China

## Introduction

The emergence of Disease X, a rapidly spreading infectious disease caused by an unknown pathogen, has raised significant global concerns. The disease’s capacity for swift mutation and cross-species transmission has amplified the urgency to understand its origins and implement effective control measures.^[1]^ Traditional methods of tracing disease outbreaks, including epidemiological surveillance and laboratory diagnostics, are struggling to address the complexities associated with this novel threat. In this context, artificial intelligence (AI) offers a promising approach to enhance disease surveillance, improve diagnostic accuracy, and predict potential risks.

AI’s ability to analyze large-scale data from diverse sources—such as genomic sequences, epidemiological records, and environmental data—provides an unprecedented opportunity to bolster efforts in tracing Disease X. Through the application of advanced algorithms and machine learning models, AI can identify data patterns, detect novel mutations in pathogens, predict disease transmission dynamics, and aid in the development of vaccines and therapeutic interventions. However, despite its considerable potential, the integration of AI into disease control efforts is not without challenges. Key issues include the quality and availability of data, the interpretability of AI models, and the need for crossdisciplinary collaboration between virologists, epidemiologists, and AI specialists.

This paper aims to review the current state of AI applications in Disease X surveillance and prevention, examine the challenges hindering its effective implementation, and propose future directions for research and development in this area.

## Disease X tracing: Background and importance

### Epidemiological features and threat of disease X

**Figure 1:**
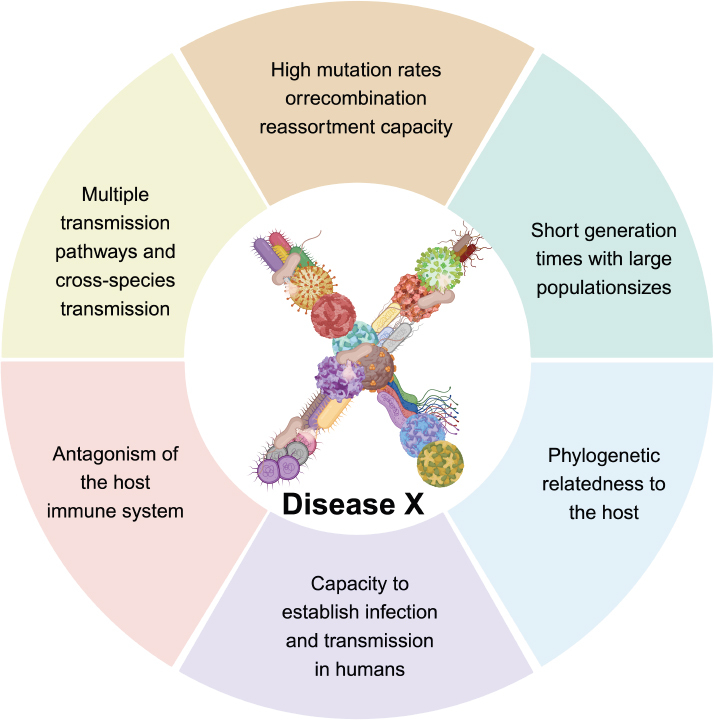
Key characteristics of Disease X highlighting its potential to emerge as a novel pathogen with pandemic risk. Features include high mutation rates, reassortment capacity, multiple transmission pathways including crossspecies transmission, short generation times, phylogenetic relatedness to the host, antagonism of the host immune system, and the ability to establish infection and transmission in humans. Created in BioRender.com.

Disease X poses a significant threat due to the unknown nature of its causative agent, coupled with the pathogen’s high mutation rates and its potential for rapid transmission across populations and regions (Figure 1).^[2]^ These factors complicate efforts to predict the disease’s trajectory and effectively control its spread. The ability to trace the origins of the disease and monitor its progression in real time is crucial for mitigating its impact.

### The role of tracing in disease control

Tracing the origin and transmission pathways of Disease X is essential for effective public health responses.^[3]^ Understanding the disease’s spread enables authorities to implement targeted interventions, identify at-risk populations, and allocate resources more efficiently. Furthermore, tracing efforts can guide the development of vaccines and therapeutic agents by revealing the pathogen’s structure and characteristics. However, the complex transmission dynamics of Disease X, coupled with its rapid evolution, render traditional tracing methods insufficient, highlighting the need for the integration of AI technologies.

## Applications of AI in disease X tracing

### Pathogen detection and identification

AI plays a pivotal role in the detection and identification of the pathogen responsible for Disease X (Supplementary Figure 1). High-throughput sequencing (HTS) technologies generate vast amounts of genomic data that require advanced algorithms for meaningful interpretation. AI models, particularly deep learning techniques such as Convolutional Neural Networks (CNNs), can be leveraged to identify specific pathogen sequences within genomic data, detect mutations, and monitor viral evolution.^[4]^ During an outbreak of Disease X, AI can dynamically track viral mutations, identifying those likely to influence transmission rates or virulence. This rapid genomic surveillance facilitates real-time public health responses.

### Epidemic prediction and spread modelling

AI techniques, including time-series analysis and Long Short-Term Memory (LSTM) networks, can be used to model the spread of Disease X based on historical infection data. These models help identify high-risk regions and populations, enabling targeted interventions. Predictive models also support resource allocation and guide containment strategies.

### Environmental and cross-species transmission predictions

Cross-species and environmental transmission present significant challenges in understanding Disease X.^[5]^ AI models can process environmental data, such as climate conditions and population density, to predict transmission risks between wildlife, livestock, and humans. By analyzing these variables, AI can forecast potential outbreaks across different regions and species, aiding in the prevention of new transmission cycles.

### Monitoring viral mutations

AI’s ability to track viral mutations is crucial in the fight against Disease X. Real-time analysis of viral genomic sequences allows for the identification of emerging variants with increased transmissibility or resistance to existing treatments.^[6]^ By continuously monitoring viral evolution, AI can offer early warnings of potential vaccine or therapeutic resistance, facilitating the timely adaptation of public health strategies.

### Vaccine and drug development

AI accelerates vaccine and drug development by optimizing antigen design, screening potential drug candidates, and predicting their efficacy. Machine learning algorithms can analyze genomic and protein structural data to identify the most immunogenic epitopes for vaccine development.^[7]^ Additionally, AI models enable the screening of large chemical libraries to identify molecules that may inhibit the pathogen, thereby shortening the drug discovery process.

## Opportunities and challenges for AI in disease X tracing

### Big data integration and model development

AI excels at integrating diverse datasets, including genomic sequences, epidemiological data, and environmental variables. Machine learning algorithms can extract valuable features from noisy or incomplete data, enhancing the accuracy and speed of disease tracing.^[8]^ However, the vast and heterogeneous nature of these datasets presents challenges in data preprocessing, integration, and interpretation.

### Real-time surveillance and response

AI models enable real-time monitoring of Disease X, allowing for the identification of trends and the prediction of future outbreaks. Automated data collection and sensor networks facilitate the rapid accumulation of information, providing valuable insights for timely public health interventions.

### Improving tracing efficiency and coverage

Traditional tracing methods are often constrained by geographic scope and limited data availability. In contrast, AI’s ability to analyze large-scale, cross-regional datasets enables the rapid tracking of Disease X across borders, offering a global perspective on its spread and fostering international cooperation in containment efforts.

## Future directions

### Data standardization and sharing

The development of standardized data-sharing platforms is essential for addressing the challenges posed by data heterogeneity. By establishing consistent data formats and protocols, international collaboration can be enhanced, enabling AI models to be built on more comprehensive datasets.^[9]^

### Explainable ai for public health decision-making

Future AI models should be designed with interpretability in mind, ensuring that their predictions are understandable and trustworthy for public health authorities.^[10]^ Hybrid models, which combine data-driven learning with domain expertise, show promise in achieving this goal.

### Cross-disciplinary research and innovation

Encouraging collaboration among AI researchers, epidemiologists, virologists, and public health experts is crucial for advancing AI applications in disease tracing. Interdisciplinary research can foster innovative solutions to the complex challenges posed by Disease X.

### Privacy-preserving technologies

The integration of privacy-preserving technologies, such as differential privacy and federated learning, is crucial for ensuring the secure handling of data used in AI models. These technologies enable data to be shared and analyzed without compromising individuals’ privacy.

## Conclusion

AI has the potential to revolutionize the tracing and control of infectious diseases like Disease X. By enabling more accurate, timely, and global disease surveillance, AI can aid in predicting outbreaks, guiding interventions, and accelerating vaccine and drug development. However, to fully realize these benefits, challenges related to data quality, model transparency, interdisciplinary collaboration, and privacy protection must be addressed. With continued research and development, AI will play an increasingly vital role in safeguarding public health against future pandemics.

### Supplementary Information

Supplementary materials are only available at the official site of the journal (www.intern-med.com).

## Supplementary Material

Supplementary Material
