# Supplementary material for: Artificial intelligence for disease X: Progress and challenges
Source: J Transl Int Med. 2025 Jan 10;12(6):534–6. doi: 10.1515/jtim-2024-0035 (PMC11720928; doi:10.1515/jtim-2024-0035)
Supplement: Supplementary file 1 — Supplementary Material [file jtim-2024-0035_sm.pdf]

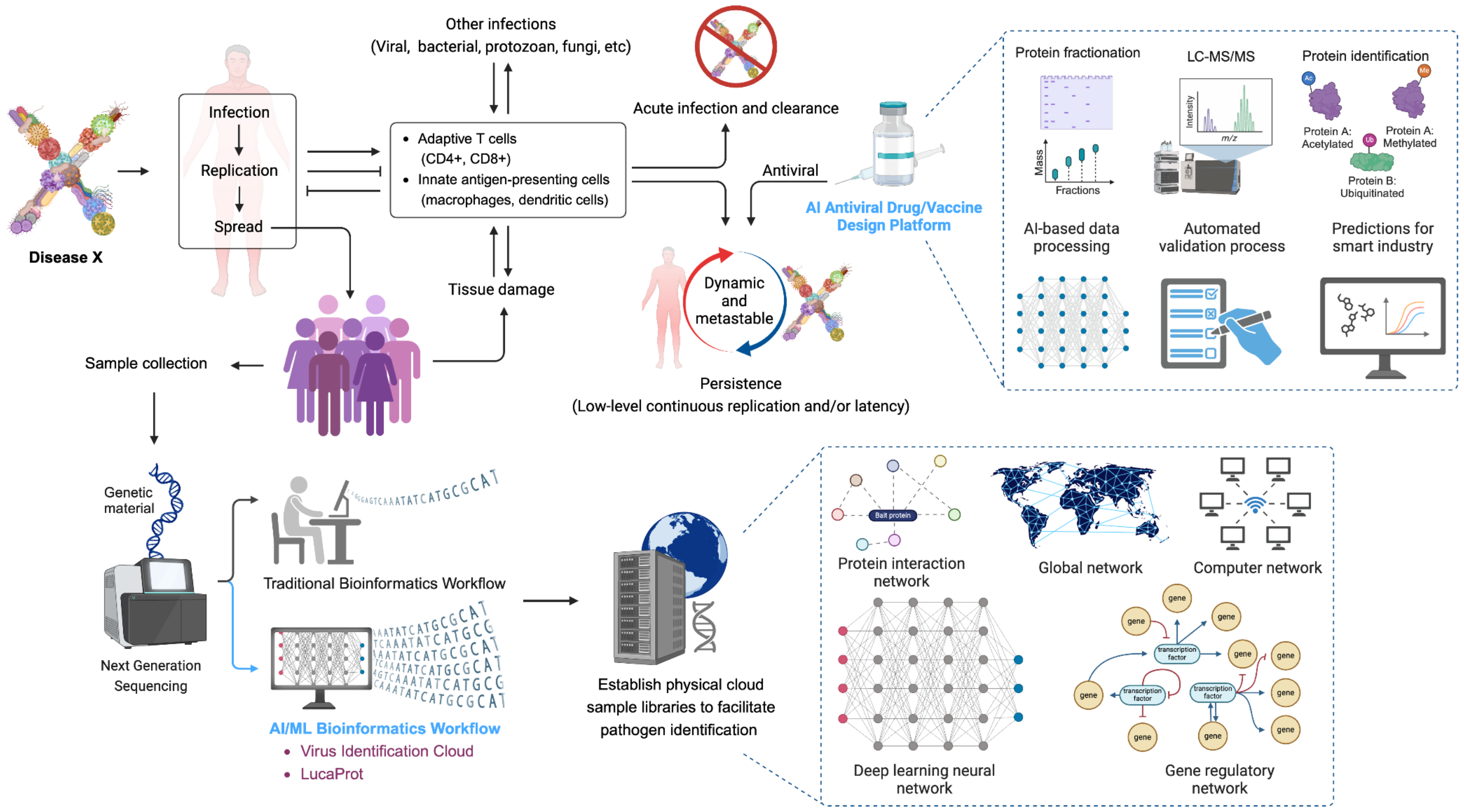
Disease X
Infection
Replication
Spread
Other infections
(Viral, bacterial, protozoan, fungi, etc)
Adaptive T cells (CD4+, CD8+)
Innate antigen-presenting cells (macrophages, dendritic cells)
Tissue damage
Acute infection and clearance
Antiviral
Dynamic and metastable
Persistence
(Low-level continuous replication and/or latency)
AI Antiviral Drug/Vaccine Design Platform
Protein fractionation
LC-MS/MS
Protein identification
Fractions
Mass
Intensity
m/z
Protein A: Acetylated
Protein A: Methylated
Protein B: Ubiquitinated
AI-based data processing
Automated validation process
Predictions for smart industry
Sample collection
Genetic material
Next Generation Sequencing
Traditional Bioinformatics Workflow
AI/ML Bioinformatics Workflow
Virus Identification Cloud
LucaProt
Establish physical cloud sample libraries to facilitate pathogen identification
Bait protein
Protein interaction network
Global network
Computer network
Deep learning neural network
gene
transcription factor
Gene regulatory network

Supplementary Figure 1. AI-driven workflow for tracing the origins of Disease X and accelerating downstream drug and vaccine development. The process integrates sample collection, next-generation sequencing, and advanced bioinformatics, with a focus on AI/ML tools such as virus identification clouds and LucaProt for pathogen identification. AI platforms streamline antiviral drug and vaccine design, leveraging dynamic infection modeling and persistence studies. Proteomics tools, including LC-MS/MS, utilize AI-based data processing and validation, supporting the discovery of protein-based therapeutic targets and global regulatory networks. Created in BioRender.com.
